# Are We Considering All the Potential Drug–Drug Interactions in Women’s Reproductive Health? A Predictive Model Approach

**DOI:** 10.3390/pharmaceutics17081020

**Published:** 2025-08-06

**Authors:** Pablo Garcia-Acero, Ismael Henarejos-Castillo, Francisco Jose Sanz, Patricia Sebastian-Leon, Antonio Parraga-Leo, Juan Antonio Garcia-Velasco, Patricia Diaz-Gimeno

**Affiliations:** 1 IVIRMA Global Research Alliance, IVI Foundation, Instituto de Investigación Sanitaria La Fe, Av. Fernando Abril Martorell 106, Torre A, Planta 1, 46026 Valencia, Spain; pablo.garcia@ivirma.com (P.G.-A.); ismael.henarejos@ivirma.com (I.H.-C.); patricia.sebastian@ivirma.com (P.S.-L.); antonio.parraga@ivirma.com (A.P.-L.); juan.garcia.velasco@ivirma.com (J.A.G.-V.); patricia.diaz@ivirma.com (P.D.-G.); 2 Department of Pediatrics, Obstetrics and Gynecology, University of Valencia, Av. Blasco Ibáñez 15, 46010 Valencia, Spain

**Keywords:** drug–drug interactions, side effects, female reproductive therapies, polypharmacology, gynecology, pharmacotherapy

## Abstract

**Background:** Drug–drug interactions (DDIs) may occur when two or more drugs are taken together, leading to undesired side effects or potential synergistic effects. Most clinical effects of drug combinations have not been assessed in clinical trials. Therefore, predicting DDIs can provide better patient management, avoid drug combinations that can negatively affect patient care, and exploit potential synergistic combinations to improve current therapies in women’s healthcare. **Methods:** A DDI prediction model was built to describe relevant drug combinations affecting reproductive treatments. Approved drug features (chemical structure of drugs, side effects, targets, enzymes, carriers and transporters, pathways, protein–protein interactions, and interaction profile fingerprints) were obtained. A unified predictive score revealed unknown DDIs between reproductive and commonly used drugs and their associated clinical effects on reproductive health. The performance of the prediction model was validated using known DDIs. **Results:** This prediction model accurately predicted known interactions (AUROC = 0.9876) and identified 2991 new DDIs between 192 drugs used in different female reproductive conditions and other drugs used to treat unrelated conditions. These DDIs included 836 between drugs used for in vitro fertilization. Most new DDIs involved estradiol, acetaminophen, bupivacaine, risperidone, and follitropin. Follitropin, bupivacaine, and gonadorelin had the highest discovery rate (42%, 32%, and 25%, respectively). Some were expected to improve current therapies (n = 23), while others would cause harmful effects (n = 11). We also predicted twelve DDIs between oral contraceptives and HIV drugs that could compromise their efficacy. **Conclusions:** These results show the importance of DDI studies aimed at identifying those that might compromise or improve their efficacy, which could lead to personalizing female reproductive therapies.

## 1. Introduction

Drug–drug interactions (DDIs) may occur when two or more drugs are co-administered to a patient. In some cases, the drugs may act synergistically to amplify the pharmacological effect, but in others, the interactions can reduce pharmacodynamic efficacy, cause adverse drug events (ADEs) [1], or have variable effectiveness [2]. DDIs are emerging as a public health concern, with a recent study estimating that DDIs account for 5% of all hospital admissions [2].

Considering the vast number of over-the-counter and prescription drugs on the market, the average American adult takes three drugs per day [3], and 22.4% of adults were dispensed with five or more drugs [4], with women more likely to take polypharmacy than men [5]. In the context of female reproductive medicine, the DDIs between contraceptives, antiretroviral drugs, i.e., for human immunodeficiency virus (HIV), and other unrelated drugs are alarming clinicians [6,7]. Although there are several ongoing studies related to DDIs on contraception [8,9,10], patients undergoing fertility treatments sometimes need to take multiple drugs to treat their complex conditions, causing potential DDIs and ADEs. Indeed, in a recent study of 440 patients with polycystic ovary syndrome (PCOS), up to nine different contraceptives and infertility drugs were co-administered, causing 26.1% of these patients to present DDIs, which required close monitoring [11]. With the uncertainty of the long-term effects of coronavirus disease 2019 (COVID-19) on women’s reproductive health [12,13], and the combination of drugs used to manage its systemic symptoms, patients with COVID-19 may similarly be at risk. Taken together, predicting DDIs could offer substantial benefits, especially for women undergoing assisted reproduction treatments (ARTs). After further validation, the identification of DDIs may allow clinicians to offer safer, more effective therapeutic strategies, while opening paths to develop new combinations of treatments [14].

Further, studying DDIs through in silico approaches based on predictive models is both cost-effective and efficient, compared to conventional in vitro or in vivo approaches. Most in silico approaches to predict new DDIs are based on the similarities between drugs (e.g., mainly chemical similarity) [15], but also their comparable side effects [16,17], known interactions with other drugs, denoted as the interaction profile fingerprint (IPF) [18], shared targets, enzymes, carriers, transporters, or molecular signaling pathways [19,20], or proximity of targets in the human interactome [21]. Indeed, Vilar et al. published a detailed methodology for integrating a reference database of known DDIs, with all the drug similarities, to significantly improve their predictions [18]. To our knowledge, drugs used in any capacity in women’s reproductive health, hereafter referred to as women’s reproductive health drugs (WRHDs), have not been assessed in-depth by these methods. Herein, we present a framework to analyze known DDIs and predict new DDIs in the context of women’s reproductive health. Thus, the novel WRHDs interactions described here cover different reproductive statuses (from preconception to menopause); associated reproductive diseases or conditions (including uterine, ovarian, and menstrual disorders); clinical application (in vitro fertilization [IVF], and ovarian stimulation); relevant diseases (HIV, COVID-19) contraceptives; and drugs not specifically used for reproduction, but potentially taken concurrently (e.g., anesthetics, related to surgical-procedures, or to treat conditions outside of reproductive health). For the first time in gynecology, this study innovatively compiled information of drugs used to treat women’s reproductive diseases/conditions, with their known DDIs (extracted from pharmacological databases), to predict novel DDIs, that can help future research for clinical decision making in gynecology, and ultimately, achieve safer and more effective treatments in women’s reproductive medicine.

## 2. Materials and Methods

### 2.1. Compilation of Approved Drugs Indicated for Women’s Reproductive Medicine

Drug data related to women’s reproductive statuses or clinical application [i.e., preconception, infertility, menopause, or IVF], diseases, and conditions (i.e., menstrual, uterine, ovarian, or other reproductive disorders) were classified according to the query terms listed in Supplemental Table S1A. Guidelines of the European Society of Human Reproduction and Embryology (ESHRE) were consulted to identify approved drugs indicated for the management of endometriosis [22], premature ovarian insufficiency (POI) [23], recurrent pregnancy loss (RPL) [24], polycystic ovary syndrome (PCOS) [25], Turner syndrome [26], ovarian stimulation [27], and oocyte retrieval procedures [28], until September 2020. To complete the list of approved WRHDs, we then consulted PubMed, the DrugBank database [29], and ClinicalTrials.gov (for drugs in phase IV clinical trials, used in women with reproductive diseases between 2013 to 2020).

### 2.2. Building the Model to Predict Drug–Drug Interactions Involving Women’s Reproductive Health Drugs

Known DDIs (n = 117,002) for each WRHD included in the study were obtained from DrugBank [29]. Six drug features were evaluated: (i) chemical structure (based on the premise that if drug A and drug B interact to produce a biological effect, then drugs with chemical similarity to drug A or drug B can produce the same effect when they are combined) [15] obtained from DrugBank; (ii) drug targets, enzymes, transporters, and carriers obtained from DrugBank (since DDIs can occur when molecular entities are shared) [20]; (iii) ADEs reported in the Side Effect Resource (SIDER) [30]; (iv) targeted biological pathways included in Kyoto Encyclopedia of Genes and Genomes (KEGG) [31]; (v) proximity of drug targets in the human interactome [32,33] from the CCSB Interactome Database; and (vi) the IPF of each drug (which assumes that if two drugs share similar interaction partners with other drugs, then these two drugs could also interact between themselves) [18].

Afterwards, the methodological protocol reported by Vilar et al., 2014, to build a prediction model was strictly followed [18]. First, we built a reference matrix (M1), which represents all the known interactions between the selected drugs described in Drugbank (i.e., interactions among 4014 selected drugs). Secondly, as aforementioned, we collected crucial drug features (chemical structure, drug targets/enzymes/transporters/carriers, adverse drug events, biological pathways, protein interactome proximity, and interaction profile fingerprints) to calculate similarities between drugs using the Tanimoto index [34]. As a result, six M2 matrices, one for each relevant drug feature, were generated. Thirdly, we combined both the interactions of drugs (M1) and the drug features (M2) to generate six new matrices (M3), each representing the predicted scores for each DDI from a different biological perspective. Some of these predicted DDIs will be known, and others will be previously unknown. Finally, we applied a principal component analysis (PCA) using the ROCR package in R [35] to integrate the predicted scores for each drug feature and to obtain a final predictive model supported by strong and high-confidence information. An illustrated example and further details are shown in the supplemental methods. Additionally, to validate the ability of our model to predict new DDIs, we assessed the integrated scores obtained after PCA for predicting the known DDIs, using AUROC with a cross-validation technique (10-fold) through the ROCR package [16,18].

Regarding establishing a threshold of confidence for the novel interactions predicted, we selected, following the recommendation of Vilar et al., the predicted scores higher than the third quartile of the distribution of all predicted scores for existing interactions. More details can be found in the supplementary methods. After filtering the predicted DDIs by this threshold of confidence, the potential biological effect of each DDI was classified according to annotations in DrugBank, assessing their potential clinical impact. Specifically, DDI effects were classified as PK, PD, and adverse effects according to the annotations from DrugBank (further details can be found in the supplemental methods). Next, we performed a descriptive study of these WRHD DDIs among themselves, and also among the IVF-specific drugs encompassed in the WRHD. Namely, we determined whether a DDI is beneficial or harmful by relying on the annotations provided by the DrugBank database and the bibliography associated with interacting drugs. Finally, we investigated possible interactions with COVID-19 and HIV drugs, and the interactions of IVF drugs with non-gynecological drugs.

## 3. Results

### 3.1. Classifying Drugs Used in Women’s Reproductive Health

Based on our searches of the ESHRE guidelines, ClinicalTrials.gov, DrugBank, and PubMed, we identified 192 WRHDs associated with 51 different reproductive conditions or diseases (Supplemental Table S1B), classified into the following categories: preconception (47 drugs), infertility and IVF (58 drugs), menopause (9 drugs), uterine diseases (83 drugs), ovarian diseases (67 drugs), menstrual disorders (17 drugs), and other reproductive disorders (73 drugs).

### 3.2. Known and Novel Drug–Drug Interactions Involving Women’s Reproductive Health Drugs

Including the 192 unique WRHDs, a total of 4014 approved drugs were retrieved from DrugBank. An exhaustive search in DrugBank yielded 117,002 known DDIs among the 192 WRHDs and the other 3822 approved drugs (Supplemental Table S2), which we then used to validate model’s performance (AUROC = 0.9876 ± 0.0149, for predicting the known DDIs with 96.6% sensitivity and 96.6% specificity) as a proof of concept for the accuracy for predicting the 2991 new ones discovered in this work (see supplemental methods for more detailed information). To highlight novel interactions, we set the threshold at the third quartile (0.7418) of the validation set. After filtering by this threshold, we obtained 2991 novel predicted interactions (Supplemental Table S3) between the WRHDs and the drugs for non-gynecological indications, a novel discovery of 2.5% with respect to the 117,002 known DDIs used in the validation set. Notably, 15 of the 192 WRHDs (7.8%) did not have any known or predicted interactions, and 8 of these were IVF drugs [i.e., luteinizing hormone, lutropin alfa, urofollitropin, menotropins, human chorionic gonadotropin (hCG) and its alpha subunit (hCGα), cetrorelix, and ganirelix]. Our findings highlight that DDIs can not only affect a variety of conditions and diseases (including uterine disorders, PCOS, and infertility), but also produce ADEs and changes in the pharmacokinetics and pharmacodynamics of WRHDs. Estradiol was distinguished as the WRHD with the most interactions from the preconception and the menopause groups; acetaminophen for menstrual disorders; bupivacaine in uterine diseases; risperidone for other reproductive diseases; and follitropin (a recombinant form of follicle-stimulating hormone) for infertility and ovarian disease groups (Table 1). On the other hand, follitropin, bupivacaine, and gonadorelin were the WRHDs with the highest DDI discovery rate (Table 2).

Regarding the interactions between WRHDs and contraceptives or HIV drugs, we predicted twelve novel DDIs for amphotericin B and three for alitretinoin. We found that three COVID-19-related drugs were under investigation to treat reproductive disorders (i.e., azithromycin for preeclampsia, ibuprofen for preeclampsia and endometriosis, and dexamethasone for PCOS). Further, we predicted that heparin (indicated for RPL) may improve the therapeutic efficacy of chloroquine and methylprednisolone (two drugs used to treat COVID-19); four novel interactions between azithromycin and antihypertensive agents [producing an extended interval between the heart contracting and relaxing (QT prolongation)]; nine between Ibuprofen and drugs indicated for gastrointestinal disorders, hypertension, diabetes, asthma, mental disorders, and inflammation; and six between dexamethasone and antihypertensives, mental disorder drugs, and antineoplastics (Supplemental Table S4).

### 3.3. Drug–Drug Interactions Predicted to Pose a Problem for Women’s Reproductive Health

Analyzing the predictions from our model, we report novel DDIs for our WRHDs with relevant clinical impact (Table 3). Patients co-administered fentanyl and follitropin may have an increased risk or severity of cardiac arrhythmia, while cyclosporine may decrease the efficacy of follitropin. In concordance with previous studies, we found that combining progesterone and estradiol might increase the risk or severity of liver damage. Further, we also found that prednisolone might accelerate the metabolism of midazolam and lidocaine, which are both used for anesthesia during oocyte retrieval procedures. Finally, we highlight an interesting prediction in which patients taking cabergoline may experience reduced estradiol metabolism during controlled ovarian stimulation.

### 3.4. Drug–Drug Interactions Predicted to Enhance the Therapeutic Efficacy of Current Women’s Reproductive Health

We identified 23 DDIs that could potentially improve the therapeutic efficacy of one drug when combined with another (Table 4). Specifically, we predicted that when vitamin D and estradiol are co-administered (e.g., in the treatment of PCOS), the accelerated metabolism of estradiol may occur. Interestingly, four of the nine drug combinations predicted to improve the efficacy of triptorelin (used for patients with PCOS) involved other drugs commonly administered to patients with PCOS [i.e., isotretinoin (indicated for PCOS-related acne), levocarnitine (L-carnitine; indicated for insulin resistance- or obesity-related PCOS), folic acid, and pyridoxine (also indicated for endometriosis)]. We also note interactions between isotretinoin and gonadorelin, and methyldopa (indicated for preeclampsia) and chlorothiazide (currently indicated for hypertension).

### 3.5. Predicted Conflicts Between IVF Drugs and Drugs for Non-Gynecological Indications

The subset of IVF drugs (n = 58) (listed in Supplemental Table S5) from the WRHDs was used to analyze the predicted interactions with the most common drugs used in the general clinical setting. We predicted 836 novel interactions between IVF drugs and drugs approved for non-gynecological indications [mainly for asthma, allergies, anti-inflammation, analgesics, sedatives, and diabetes (Table 5A and Supplemental Table S5)]. Specifically, follitropin was the IVF drug with the most predicted DDIs (n = 82). Estradiol mainly interacted with drugs related to asthma and allergy (n = 9) or diabetes (n = 3), while cabergoline mainly interacted with anti-inflammatories, analgesics, and sedatives (n = 21). Networks showcasing the distribution of normalized interactions between WRHDs or IVF drugs and their therapeutic indications are shown in Table 5B and Table 5C, respectively. Estradiol had the most interactions between IVF drugs (n = 7), while bupivacaine had the most among all WRHDs (n = 21). Drugs indicated for uterine disorders showed the highest number of DDIs with the rest of the WRHDs (n = 48), whereas drugs used for infertility had the largest number of DDIs with IVF drugs (n = 20). Specific data for these interactions is shown in Supplemental Table S5.

## 4. Discussion

To our knowledge, this is the first study to use a predictive model for DDIs in women’s reproductive health, which could potentially improve the clinical management of ART treatments. In addition to summarizing known interactions at a systematic level, we adapted a robust prediction model to discover unknown DDIs that can compromise the efficacy and safety of current ART treatments, or, alternatively, improve their effectiveness [14]. Individually analyzing the DDIs allowed us to elucidate their potential clinical implication and advise clinicians to observe the reduced efficacy, unknown ADEs, or therapeutic benefits, resulting from the combination of drugs used in female reproductive medicine, and set the stage for clinical trials that aim to improve the treatment of women’s diseases/conditions.

The predictive model was built using the biological and pharmacological data of 4014 approved drugs retrieved from DrugBank. This data encompassed chemical structures, known ADEs, shared targets, carriers, enzymes, transporters, pathways, interactome data, and interaction profiles [15,16,17,18,19,20], following the methodology proposed by Vilar et al., who were pioneers in this type of study [18]. The capacity of our model to accurately predict the 117,002 known interactions for the 4014 drugs from DrugBank demonstrates the reliability of the novel interactions we predicted in this study.

### 4.1. Identifying Clinically Relevant Drug–Drug Interactions to Aid ART Practitioners

Our model highlighted follitropin (indicated for ovarian stimulation) at the forefront of the novel DDIs discovery. However, considering the normalized proportion of predicted interactions with drugs indicated for non-gynecological disorders, we found that IVF drugs interact mainly with asthma and allergy drugs, followed closely by drugs for diabetes (both with estradiol coming on top) and anti-inflammatories, analgesics, and sedative drugs (in the case of cabergoline). Taken together, our findings reinforced that female ART drugs can interact with other medications indicated for non-gynecological disorders.

Upon close examination of the predicted interactions, we report different recommendations for drug combinations according to the effect of the predicted interaction. We predicted nine positive DDIs that can increase the efficacy of triptorelin suggested for the treatment of endometriosis [36,37], which is often co-administered with oral contraceptives. We also found a potential synergistic effect for the combination of isotretinoin and gonadorelin [38,39], which are recommended for the treatment of PCOS-related acne when oral contraceptives are contraindicated. According to our results, the interaction between these two drugs would enhance the efficacy of isotretinoin. We also predicted a synergistic interaction between triptorelin and levocarnitine. Recent studies suggest that levocarnitine improves pregnancy rates and alleviates symptoms of ovarian dysfunction [40]. Therefore, the addition of triptorelin to levocarnitine-based treatment regimens merits prospective clinical validation in patients with endometriosis. Further, we predicted that vitamin D promotes the metabolism of estradiol [41,42]. Women with unbalanced levels of endogenous estradiol are at risk of developing endometriosis [43]. Therefore, a prospective validation of vitamin D for the treatment of endometriosis warrants attention. Finally, we predicted a positive interaction of methyldopa (an antihypertensive agent used for preeclampsia) and chlorothiazide (currently indicated for hypertension, but not ARTs) [44]. Interestingly, chlorothiazide has enhanced the effectiveness of other antihypertensive drugs [29], but its combination with methyldopa has not yet been evaluated clinically, especially in the context of preeclampsia.

Alternatively, based on the possible adverse DDIs we predicted, we warn that the combination of fentanyl (used as an anesthetic during oocyte retrieval procedures) [45] and follitropin (used for ovarian stimulation) [46] may increase the risk or severity of cardiac arrhythmia. Although the administration of these two drugs is not concurrent per se, a slower metabolism of follitropin may overlap with fentanyl administration. We also validate that cabergoline slows the metabolism of estradiol, supporting the findings by Lin et al. [47], which argued that the addition of cabergoline to GnRH antagonist protocols may be detrimental to uterine receptivity by maintaining high serum estradiol levels, thus supporting the strategy of embryo cryopreservation following a GnRH antagonist protocol [48,49]. Further, despite the widespread use of estradiol and progesterone in female reproductive medicine [50,51], the risk of liver damage when administering both drugs has been previously described (possibly through elevated levels of liver aminotransferases) [52,53] and of developing cholestasis or other hepatic conditions, even if sometimes this risk is known and accepted [54,55,56,57]. We predicted the potential increase in this risk, reinforcing the validity of our drug–drug interactions predictions. Regarding treatments for reproductive conditions, most interactions predicted as harmful were found between drugs indicated for PCOS.

### 4.2. Clinical Implications

This study highlights the advantages of using accurate predictive models to report unknown DDIs, which could allow advanced post-market pharmacovigilance. Moreover, clinicians could take these results into consideration for identifying unreported ADEs and/or potential altered clinical effectiveness during their routine clinical practice. Additionally, these predicted new interactions will allow clinicians to propose alternative drug combinations that may improve the effectiveness and safety of currently available treatments, and ultimately, patients’ fertility or reproductive health. Finally, these results could be the basis for the development of clinical trials aimed at pinpointing important DDIs that might worsen women’s fertility outcomes, hindering the hard process of ARTs that has been proven to be mentally and physically demanding for women.

### 4.3. Research Implications

This is the first study to analyze and compile a list of known DDIs for women’s reproductive health, and predict compromising and synergistic DDIs, not only increasing awareness of DDIs in the context of women’s reproductive health but also identifying research gaps that can be addressed by future clinical studies aimed to develop novel treatments for female patients undergoing ARTs.

### 4.4. Strengths and Limitations

Between 2009 and 2017, the US Food and Drug Administration (FDA) approved 302 new drugs [58], but their post-market pharmacovigilance was limited with respect to evaluating possible interactions between ART drugs and other previously approved drugs in clinical use. In this regard, the pharmaceutical industry, regulatory agencies, public health services, and patients would benefit from the development of robust prediction models to discover novel DDIs. However, the model is limited to what is stored in the databases consulted, with many drugs lacking information about their targets, ADEs, and more; the incompleteness of the human interactome. Additional factors known to affect the occurrence of DDIs—including drug dosage, pharmaceutical formulation, and the influence of genetic polymorphisms on individual drug dosage (pharmacogenetics)—were not incorporated into the DDI prediction model due to their absence in the available data sources and the assumption that the databases are well curated, among other limitations. Furthermore, prospective studies are needed to validate the DDIs predicted by our model.

Nonetheless, this study sheds light on DDIs relevant to women’s reproductive health, revealing previously unknown DDIs that could compromise the efficacy of ART treatments or boost the therapeutic effects of drugs, such as follitropin or triptorelin. We acknowledge that further experimental and clinical evidence is needed to validate our predictions; however, we note that our prediction model was highly sensitive when evaluating currently known DDIs, making it a promising tool for precision medicine. Indeed, the information generated from this study could be implemented in institutional computerized prescription systems that alert clinicians of potential conflicts when the medication is ordered for clinical observational studies to modify current practices.

## 5. Conclusions

This study innovatively integrated drug data from different biological, chemical, and clinical sources into a prediction model in women’s reproductive health. The model discovered 2.5% of new DDI potential interactions in the context of women’s reproductive health. Our findings particularly distinguished DDIs that could compromise or boost the efficacy of PCOS treatments, along with novel interactions that may affect contraceptive use, HIV, or COVID-19 treatments. Despite the need to experimentally validate the predicted DDIs, these findings elucidate the complexities of drug interactions and provide opportunities for clinically relevant studies.

## Figures and Tables

**Table 1:** Top 10 female reproductive drugs by highest discovery rate. IVF, in vitro fertilization.

	Reproductive Statuses			Reproductive Conditions			
Nº	Preconception	Menstrual Disorders	Infertility & IVF	Ovarian Diseases	Uterus Diseases	Other Reproductive Diseases	**Menopause**
1	Estradiol	Acetaminophen	Follitropin	Follitropin	Bupivacaine	Risperidone	Estradiol
2	Etonogestrel	Tramadol	Estradiol	Estradiol	Follitropin	Oxycodone	Conjugated estrogens
3	Progesterone	Diclofenac	Cabergoline	Prednisolone	Estradiol	Estradiol	Progesterone
4	Segesterone acetate	Naproxen	Prednisolone	Simvastatin	Triptorelin	Levobipivacaine	Estradiol valerate
5	Medroxyprogesterone acetate	Desogestrel	Codeine	Triptorelin	Morphine	Prednisone	Comifene
6	Mifepristone	Leuprolide	Triptorelin	Etonogestrel	Acetaminophen	Lovastatin	Vitamin D
7	Megestrol acetate	Dexketoprofen	Acetaminophen	Progesterone	Conjugated estrogens	Acetaminophen	
8	Norethisterone	Levonogestrel	Testosterone	Estradiol acetate	Etonogestrel	Conjugated estrogens	
9	Desogestrel	Piroxicam	Progesterone	Medroxyprogesterone acetate	Progesterone	Etonogestrel	
10	Ketorolac	Ethinylestradiol	Meperidine	Topiramate	Medroxyprogesterone acetate	Warfarin	

**Table 2:** Discovery rate of drug–drug interactions for female reproductive drugs. Known DDIs described correspond to the known DDIs obtained from the DrugBank database. Known DDIs predicted correspond to the known DDIs that were predicted by the model. New DDIs predicted correspond to those original new DDIs predicted by the model with a predicted score above the third quantile. The Discovery rate represents the proportion between new predicted drug–drug interactions and already known interactions described in the DrugBank database. DDIs, drug–drug interactions.

Drug	Known DDIs Described	Known DDIs Predicted	New DDIs Predicted	Discovery Rate %
Follitropin	123	122	89	42.18
Bupivacaine	523	505	247	32.03
Gonadorelin	3	3	1	25.00
Segesterone acetate	201	198	36	15.19
Prilocaine	38	26	6	13.95
Triptorelin	341	340	50	12.82
Oxycodone	834	832	102	10.92
Examestane	46	45	5	10.00
Risperidone	1113	1110	116	9.46

**Table 3:** Interactions predicted to pose clinical conflicts. Eleven previously unknown drug–drug interactions that pose potential clinical conflicts. Drugs currently used for non-gynecological indications are highlighted in bold. COS, controlled ovarian stimulation; HRT, hormone replacement therapy; IVF, in vitro fertilization; PCOS, polycystic ovary syndrome; RM, recurrent miscarriage; RPL, recurrent pregnancy loss.

Drug Interaction	Drug Indications	Predicted Effect	Extrapolation
Somatotropin-Prednisolone	Infertility-RM	Reduced efficacy of prednisolone.	Both somatotropin and prednisolone are corticosteroids used in the improvement of endometrial receptivity.
Progesterone-Estradiol	HRT-HRT	Augmented risk or severity of liver damage.	Co-administration should be considered carefully to avoid exacerbating liver damage.
Follitropin-Fentanyl	IVF-IVF	Augmented risk or severity of cardiac arrhythmia.	The use of alternative anaesthetics may avoid unnecessary complications.
Follitropin-**Cyclosporine**	IVF-Immunosuppressor	Reduced efficacy of follitropin.	The efficacy of ovarian stimulation may be compromised when patients are additionally taking cyclosporine to treat inflammatory disorders.
Prednisone-**Methylprednisolone hemisuccinate**	RPL-COVID-19	Augmented risk or severity of adverse effects.	Extra pharmacovigilance is recommended for patients with COVID-19.
Follitropin-Simvastatin	IVF-PCOS	Reduced efficacy of follitropin.	The efficacy of ovarian stimulation may be compromised.
Lidocaine-Prednisolone	Oocyte Retrieval-PCOS	Accelerated metabolism of lidocaine.	Prednisolone has been applied to reduce androgen concentrations in PCOS women before oocyte retrieval. Lidocaine is used for pain relief during oocyte retrieval. Residual prednisolone may hinder pharmacodynamics of lidocaine.
Estradiol-Liraglutide	PCOS-PCOS	Poor absorption of estradiol reduces serum concentration, and potentially, its efficacy.	In PCOS treatment regimes, estradiol is administered in the form of ethinyl estradiol tablets, to improve hormonal and lipid profiles. Liraglutide is administered for weight loss before starting ovarian stimulation.
Follitropin-Midazolam	COS-Oocyte Retrieval	Reduced efficacy of follitropin.	The efficacy of ovarian stimulation may be compromised.
Cabergoline-Estradiol	COS-COS	Slow metabolism of estradiol.	Elevated estradiol levels interfere with uterine receptivity.
Midazolam-Prednisolone	Oocyte Retrieval-PCOS	Accelerated metabolism of midazolam.	Prednisolone is used to reduce androgen concentrations in PCOS women before oocyte retrieval, while midazolam is an anaesthetic administered during oocyte retrieval procedures. Residual prednisolone may reduce period of pain relief.

**Table 4:** Interactions predicted to enhance pharmacological efficacy. Twenty-three previously unknown interactions that enhance the pharmacological efficacy of women’s reproductive health drugs. Drugs currently used for non-gynecological indications are highlighted in bold. ART, assisted reproduction technology; HRT, hormone replacement therapy; PCOS, polycystic ovary syndrome; RPL, recurrent pregnancy loss.

Drug Interaction	Drug Indications	Predicted Effect	Extrapolation
Estradiol-Vitamin D	PCOS-PCOS	The metabolism of estradiol can be increased when combined with vitamin D.	Administration of vitamin D may reduce the risk of breast cancer in patients at risk due to high natural levels of estradiol.
Triptorelin-Inositol		The therapeutic efficacy of inositol can be increased.	Current PCOS treatments can potentially be improved.
Isotretinoin-Triptorelin		The therapeutic efficacy of triptorelin can be increased.	Current PCOS treatments can potentially be improved, especially for patients affected by PCOS-related acne and that have contraindications for the use of oral contraceptives.
Levocarnitine-Triptorelin			Current PCOS treatments can potentially be improved.
Folic acid-Triptorelin			
**Practolol**-Triptorelin	Cardiac Arrhythmia-PCOS		
Isosorbide mononitrate-Triptorelin	Pregnancy Loss-PCOS		
Cefazolin-Triptorelin	Uterine Bleeding-PCOS		
Etonogestrel-Triptorelin	Contraceptive-PCOS		
Megestrol acetate-Triptorelin	Contraceptive-PCOS		
Pyridoxine-Triptorelin	Endometriosis-PCOS		
Triptorelin-Dienogest	PCOS-Endometriosis	The therapeutic efficacy of dienogest can be increased.	Both are proven to be effective in the treatment of endometriosis symptoms and after surgery.
**Chlorothiazide**-Methyldopa	Hypertension-Preeclampsia	The therapeutic efficacy of methyldopa can be increased.	While not used in ARTs, chlorothiazide has been shown to enhance the effects of other antihypertensive agents.
**Moxifloxacin**-Cabergoline	Antibiotic-Infertility	The therapeutic efficacy of cabergoline can be increased.	Current fertility treatments can potentially be improved.
Heparin-**Fluoxymesterone**	RPL-Hypogonadism	The therapeutic efficacy of heparin can be increased.	Current RPL treatments can potentially be improved.
**Levallorphan**-Diazepam	Depression-Fertility Issues	The therapeutic efficacy of diazepam can be increased.	Current fertility treatments can potentially be improved.
Gonadorelin-Isotretinoin	Infertility-PCOS	The therapeutic efficacy of isotretinoin can be increased.	Current PCOS treatments can potentially be improved, especially for patients affected by PCOS-related acne and that have contraindications for the use of oral contraceptives.
Enalapril-**Hydrochlorothiazide**	Preeclampsia-Hypertension	The therapeutic efficacy of enalapril can be increased.	Current preeclampsia treatments can potentially be improved.
Prednisone-Enoxaparin	RPL-ARTs	The therapeutic efficacy of enoxaparin can be increased	Current ART treatments can potentially be improved.
Prednisone-Bemiparin	RPL-ARTs	The therapeutic efficacy of bemiparin can be increased.	
**Methazolamide**-Norethisterone	Glaucoma-Endometriosis	The therapeutic efficacy of norethisterone can be increased.	Current endometriosis treatments can potentially be improved.
Furosemide-**Fluticasone furoate**	Preeclampsia-Asthma	The therapeutic efficacy of furosemide can be increased.	Current preeclampsia treatments can potentially be improved.
**Methazolamide**-Ulipristal	Glaucoma-Contraceptive	The therapeutic efficacy of ulipristal can be increased.	Current contraception methods can potentially be improved.

**Table 5:** Discovery rate of novel DDIs in IVF and women’s reproductive health drugs. (**A**) DDI discovery rate in IVF drugs by non-gynecological drug group. (**B**) DDI discovery rate in WRHD by women’s reproductive health drug group. (**C**) DDI discovery rate in IVF drugs by IVF drug group. Table shows the original novel DDIs predicted by the model with a predicted score above the third quantile, the number of drugs of each group in which novel DDIs have been prescribed, and the discovery rate of novel DDIs (Novel DDIs/Number of drugs) in each drug group. Known DDIs described correspond to the known DDIs obtained from the DrugBank database. Known DDIs predicted correspond to the known DDIs that were predicted by the model. DDIs, drug–drug interactions; HRT, hormone replacement therapy; IVF, in vitro fertilization; PCOS, polycystic ovarian syndrome; RPL, recurrent pregnancy loss.

**(A)**					
**Drug group**	**Novel DDIs**	**Number of drugs**	**Discovery rate of novel DDIs**	**Known DDIs described**	**Known DDIs predicted**
Alimentary	17	11	1.6	126	123
Anti Inflammatory, Analgesics, Sedatives	195	79	2.5	1183	1177
Anti-infectives	76	50	1.5	816	799
Anticonvulsants, Epilepsy	10	8	1.3	211	211
Antidepressives, Anxiolytics	53	27	2	621	621
Antineoplastics	88	49	1.8	927	907
Asthma, Allergy	75	26	2.9	315	310
Cardiovascular agents, Antihypertensives, Anticholesteremic Agents	126	66	1.9	1108	1089
Contrast agent	11	10	1.1	142	107
Diabetes	10	4	2.5	72	72
Gastrointestinal problems	32	17	1.9	245	241
Insomnia	14	8	1.8	168	168
Mental illness	33	16	1.3	279	276
Ophthalmological	14	11	1.3	61	61
Parkinson	11	5	2.2	72	72
Sex hormones	20	15	1.3	277	276
**(B)**					
**Women’s reproductive health drug group**	**Novel DDIs**	**Number of drugs**	**Discovery rate of novel DDIs**	**Known DDIs described**	**Known DDIs predicted**
PCOS	27	10	2.7	158	157
Preeclampsia	1	1	1	0	0
Infertility	11	9	1.2	14	14
Contraceptives	17	10	1.7	19	19
Menstrual disorders	3	2	1.5	3	3
Uterine disorders	48	26	1.9	326	326
RPL	15	10	1.5	48	48
**(C)**					
**IVF drug group**	**Novel DDIs**	**Number of drugs**	**Discovery rate of novel DDIs**	**Known DDIs described**	**Known DDIs predicted**
PCOS	6	6	1	102	101
Infertility	20	12	1.7	404	398
Contraceptives	3	3	1	50	50
Uterine disorders	5	5	1	93	93
Ovulation disorders	1	1	1	11	11
HRT	2	2	1	102	101
RPL	4	3	1.3	49	49

## Data Availability

Data is contained within the article or supplementary material.

## Supplementary Materials

The following supporting information can be downloaded at: https://www.mdpi.com/article/10.3390/pharmaceutics17081020/s1, Table S1. Classification of reproductive categories and drugs currently used in women’s reproductive health. (A) Keywords used to search the ESHRE guidelines, DrugBank, and ClinicalTrials.gov were classified into seven reproductive categories. (B) A total of 192 drugs currently prescribed for female reproduction indications were retrieved from ClinicalTrials.gov, DrugBank, ESHRE guidelines, and PubMed. Drugs were classified into one or more of the corresponding reproductive categories presented in (A), according to the associated keywords in the ESHRE guidelines or ClinicalTrials.gov. COS, controlled ovarian stimulation; POI, premature ovarian insufficiency; POF, premature ovarian failure; PCOS, polycystic ovary syndrome; RPL, recurrent pregnancy loss. Table S2. Interactions involving female reproductive drugs. The 117,003 known interactions used to validate the prediction model are shown, along with their predicted score, the described effect in DrugBank, and which drug is involved in female reproduction. Table S3. Interactions predicted with drugs not employed in assisted reproduction, between female reproductive drugs and IVF drugs. The 2991 predicted interactions studied are shown, along with their predicted score, the described effect in DrugBank, which drug is involved in female reproduction, which is the predicted effect, in which ESHRE guidelines the drugs are recommended, in which ClinicalTrials.gov study conditions are employed, and the predicted interaction classification according to DrugBank. Table S4. Predicted interactions between drugs used for women’s reproductive health and drugs used to treat HIV and COVID-19. Our model predicted fifteen and twenty-three interactions between drugs used for women’s reproductive health (WRHDs) and drugs approved to treat human immunodeficiency virus (HIV) and coronavirus disease 2019 (COVID-19), respectively. The predicted effect of each interaction is listed. DDI = drug–drug interaction. Table S5. Predicted interactions between non-gynecological drugs and IVF drugs, between women’s reproductive health drugs, and between IVF drugs. The table shows different sheets with the 58 drugs currently prescribed in IVF treatments, DDIs between non-gynecological drugs and IVF drugs, between women’s reproductive health drugs, and between IVF drugs. In addition, the normalized proportion of DDIs of drugs within each group is shown. hrt = Hormone replacement therapy; IVF = In vitro fertilization; pcos = Polycystic ovary syndrome; rpl = recurrent pregnancy loss; WRHDs = Women’s reproductive health drugs. Figure S1. Overview of the study design. Drugs and the already known DDIs were gathered from DrugBank database. Drug features from those drugs were also obtained from DrugBank database. Both information was combined and a prediction model was used to predict DDIs. Finally, predicted DDIs were validated using known DDIs. DDIs, drug-drug interactions. Figure S2. Generation of prediction model. Step 1: Matrix M1 represents all known drug–drug interactions extracted from DrugBank, while matrix M2 contains similarity scores between drugs based on a selected biological feature. Step 2: Matrices M1 and M2 are multiplied to generate an asymmetric matrix M12, where each cell contains a predicted interaction score. To ensure symmetry, M12 is compared with its transpose, and the maximum value between each corresponding pair of cells is selected. Step 3: Finally, M3 matrix is obtained, which contains the final predicted scores for each interaction between drug pairs. Figure S3. The distribution of scores for known DDIs. Most known interactions scored between 0.6-0.8. The third quartile (0.7418) was chosen to filter out all interactions calculated by the algorithm (over ~8,000,000 predicted interactions), to set as novel discovery.
