# Supplementary material for: Are We Considering All the Potential Drug–Drug Interactions in Women’s Reproductive Health? A Predictive Model Approach
Source: Pharmaceutics. 2025 Aug 6;17(8):1020. doi: 10.3390/pharmaceutics17081020 (PMC12389331; doi:10.3390/pharmaceutics17081020)
Supplement: Supplementary file 1 [file pharmaceutics-17-01020-s001.zip › Supplemental Methods.docx]

Study Design

An overview of the study design is shown in **Figure S1**. Briefly, drugs studied in this work were obtained from DrugBank database (see https://go.drugbank.com/) together with their known drug-drug interactions (DDIs). Additionally, a set of relevant drug features such as chemical structure, interaction profile fingerprint, side effects, pathways, target enzymes, carriers and transporters, and closeness in human interactoma were also obtained from DrugBank database. Next, all this information was combined and a prediction model previously developed by Vilar et al. (2014) was used for predicting DDIs. Finally, predicted DDIs were validated by using already known DDIs.


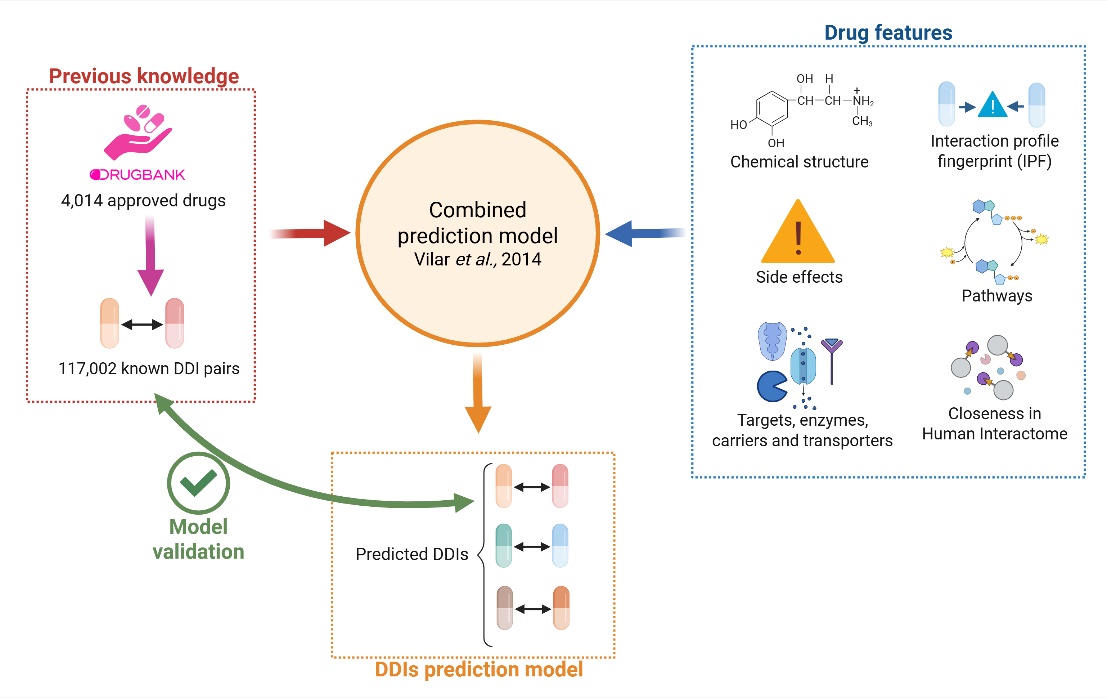


**Figure S1. Overview of the study design.** Drugs and the already known DDIs were gathered from DrugBank database. Drug features from those drugs were also obtained from DrugBank database. Both information was combined and a prediction model was used to predict DDIs. Finally, predicted DDIs were validated using known DDIs. DDIs, drug-drug interactions.

Model creation

Our approach strictly follows the established methodology developed by Vilar et al. (2024), which was used to predict new DDI’s for women’s reproductive health drugs (WRHDs). The algorithm is defined in four different steps:

1. We created a binary matrix M1 containing a total of 4,014 drugs found in DrugBanK, which represented all the known DDIs among them. Therefore, **M1** is a 4,014 × 4,014 matrix, with both rows and columns corresponding to individual drugs. Each cell in the matrix indicates whether the interaction exists, which will be 1 if the drugs have a known interaction and 0 otherwise. In total, 117,002 knows DDI pairs were identified out of the possible 16,112,196 interactions represented in the matrix (4,014 x 4,014). These interactions included those DDIs coming from manually 192 known WRDHDs.
2. For each of six drug features selected to develop the study (chemical structure, drug targets/enzymes/transporters/carriers, adverse drug events, biological pathways, protein interactome proximity, and interaction profile fingerprint), we generated another matrix representing the similarities between drugs calculated through Tanimoto index. As a result, a total of six matrices M2 (4,014 x 4,014) were created, which contained specific scores for each drug feature.
3. To generate predicted scores using the drugs interaction from matrix M1 and drug feature from matrix M2, we multiply both matrix and performed a symmetric normalization to obtain a final matrix M3 for each drug feature, which contains the predicted scores for each DDI.
4. To integrate all predicted scores obtained from a different biological perspective (M3 matrices) we applied a principal component analysis (PCA) to obtain a single integrated score for each DDI, which is supported by all the drug features previously calculated.

To illustrate the algorithm, we provided a detailed example using three clinically relevant WRDHDs: estrogen, progesterone, and gonadorelin (**Figure S2**). In this case, the matrix M1 (**Figure S2, STEP1**) represents that estrogen has a known interaction with gonadorelin, but no known interaction with progesterone. Additionally, the matrix M2 contained similarity scores based on different drug features. To ease the visualization, this example focuses only on chemical structure (**Figure S2, STEP1**). As indicated, the matrix M2 shows a moderate similarity between estrogen and progesterone (0.7) and a high similarity between progesterone and gonadorelin (0.9) (**Figure S2, STEP1**). To calculate the predicted scores, we first multiply M1 by M2 to obtain M12, retaining the **maximum value in each cell** to prioritize the most plausible interaction (**Figure S2, STEP2**). For instance, the predicted score of estrogen-progesterone pair was 0.9, which is the highest value of the multiplication. Subsequently, because matrix M12 is not symmetric (is not mirrored across the main diagonal), we compare M12 with its transpose and, for each cell, the maximum value is selected. This ensures that the predicted interaction between drug i (e.g estrogen) and drug j (e.g. progesterone) is the same as between j and i. As a result, we obtained our M3 matrix, which contains predicted scores for each DDI according to the drug feature illustrated in this example (**Figure S2, STEP 3**). The algorithm predicts an interaction between estrogen and progesterone with a score of 0.9, based on the following reasoning: estrogen is known to interact with gonadorelin (confirmed in M1), and gonadorelin shares high chemical similarity with progesterone (0.9 in M2). Based on this indirect but biologically meaningful relationship, the algorithm predicts a potential interaction between estrogen and progesterone with a value of 0.9 (**Figure S2, STEP 3**).


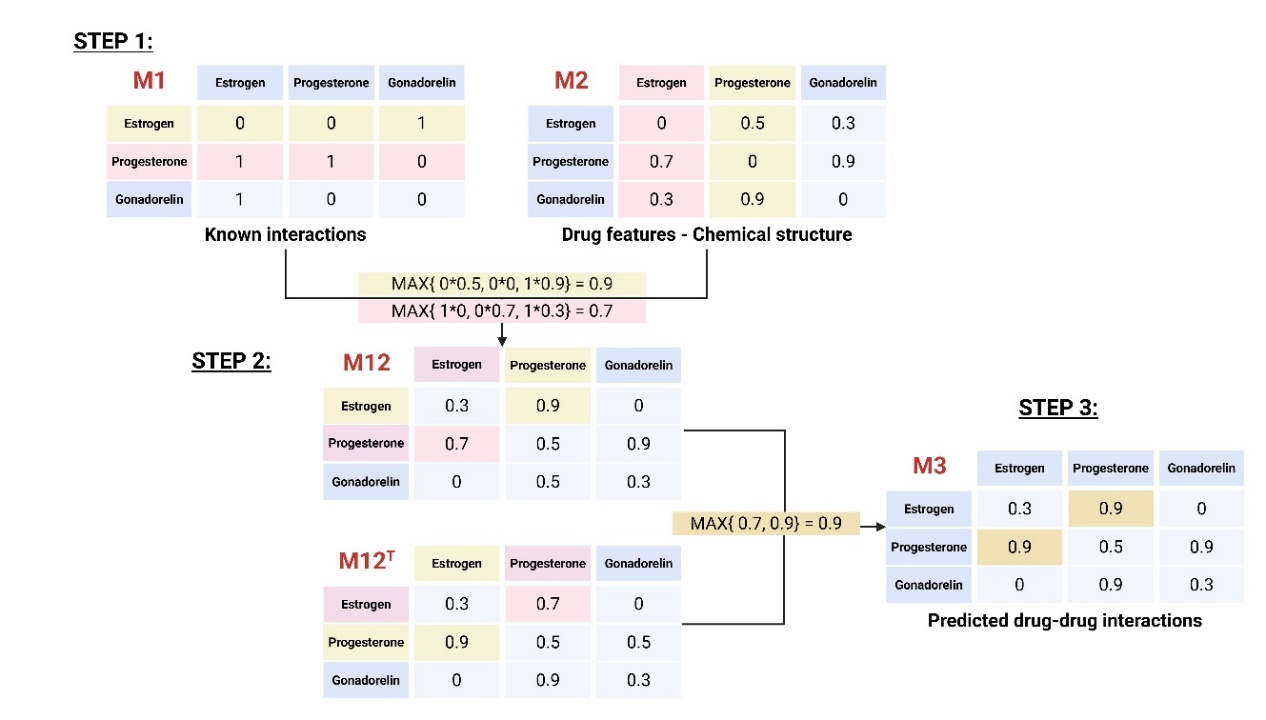


**Figure S2. Generation of prediction model. Step 1:** Matrix M1 represents all known drug–drug interactions extracted from DrugBank, while matrix M2 contains similarity scores between drugs based on a selected biological feature. **Step 2:** Matrices M1 and M2 are multiplied to generate an asymmetric matrix M12, where each cell contains a predicted interaction score. To ensure symmetry, M12 is compared with its transpose, and the maximum value between each corresponding pair of cells is selected. **Step 3:** Finally, M3 matrix is obtained, which contains the final predicted scores for each interaction between drug pairs.

Model evaluation

To evaluate whether our prediction model based on different drug features was robust and predicted accurately, we calculated the area under the ROC curve (AUROC). For this purpose, we labelled our DDIs as true positive or false positive, according whether the interaction have been previously reported in DrugBank. Then, we applied a cross-validation with 10 fold, where each DDI is used once as a test sample and nine times as training data, serving as a regularization technique to prevent overfitting. The final AUROC was obtained by averaging the AUROC values across all ten folds, providing a robust metric of the model’s overall performance.

Threshold selection

For selecting a robust threshold to report the novel 2,991 interactions, we followed Vilar et al.'s (2014) protocol recommendation, establishing our confidence threshold for new discovery among the ~8,000,000 predicted interactions (excluding the known 117,002) at the third quartile of the distribution of scores for the described set (0.7418) (**Figure S3**). This threshold ensures that only predicted interactions scoring in the top 25% of known interaction scores are considered novel discoveries. Thus, after applying our confidence threshold, we identified 2,991 novel predicted DDIs, representing a 2.5% increase over known interactions (2,991/117,002 * 100).


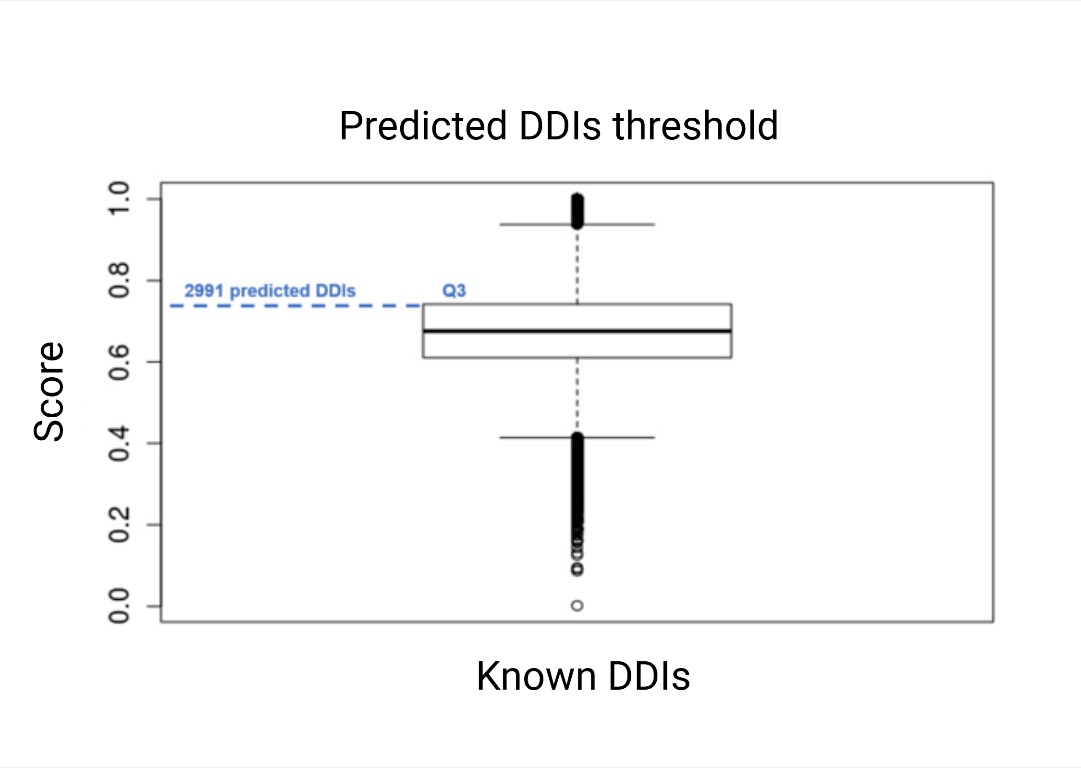


**Figure S3**. The distribution of scores for known DDIs. Most known interactions scored between 0.6-0.8. The third quartile (0.7418) was chosen to filter out all interactions calculated by the algorithm (over ~8,000,000 predicted interactions), to set as novel discovery.

Classification of DDI effects

DDI effects were classified as pharmacokinetic (PK), pharmacodynamic (PD), or side effects for simplification purposes, according to the annotations provided by the DrugBank database. Briefly, we made the following assumptions: DDIs were classified as PD when they resulted in variability in the efficacy of a drug; as PK when the DDI led to a variation in the concentration of a drug in the body (due to changes in metabolism, absorption, or excretion); and as adverse effects when the interaction caused a negative effect on the organism. An illustrative example is presented:

- PK: Drug X may decrease/increase the excretion rate (or absorption, or metabolism, or serum concentration) of drug Y. (classified as PK)
- PD: The therapeutic efficacy of drug X can be decreased/increased when used in combination with drug Y.
- Side effects: The risk of … can be increased when drug X is combined with drug Y.

Next, we performed a descriptive study of these WRHDs DDIs among themselves, and also among the IVF-specific drugs encompassed in the WRHDs. Namely, we determine whether a DDI is beneficial or harmful, by relying on the annotations provided by the DrugBank database and bibliography associated to interacting drugs. For instance, heparin is known to interact with chloroquine, resulting in an enhancement of chloroquine's therapeutic effect, as reported in the DrugBank database. If a drug (X) demonstrates a high similarity score to heparin—calculated by our model based on integrated parameters such as chemical structure, shared adverse effects, and others—it can be inferred that drug (X) is also likely to interact with chloroquine in a similar manner, potentially enhancing its therapeutic effect. This inference is grounded in the principle that structurally and functionally similar drugs tend to exert comparable pharmacological effects. Finally, we investigated possible interactions with COVID-19 and HIV drugs, and interactions of IVF drugs with non-gynecological drugs. It is important to emphasize that the source data and the effects described in DrugBank are extracted from FDA-approved drug labels, making this information curated and reliable.
